# SERUM SOLUBLE ALPHA-KLOTHO AND COGNITIVE FUNCTIONING IN OLDER ADULTS AGED 60 AND 79

**DOI:** 10.1093/geroni/igad104.1274

**Published:** 2023-12-21

**Authors:** Song Ge, Fanghong Dong, Chong Tian, Jingkai Wei

**Affiliations:** University of Houston-Downtown, Houston, Texas, United States; Hebei University, Baoding, Hebei, China (People’s Republic); Huazhong University of Science and Technology, Wuhan, Hebei, China (People’s Republic); University of South Carolina, Columbia, South Carolina, United States

## Abstract

**Objectives:**

We aimed to examine the association of serum soluble alpha-klotho with cognitive functioning among older adults using a nationally representative sample of U.S. older adults. Method: A total of 2,173 U.S. older adults aged 60-79 years in the National Health and Nutrition Examination Survey 2011 to 2014 were included. Serum soluble alpha-klotho was measured in the laboratory. Cognitive function was measured using the Consortium to Establish a Registry for Alzheimer’s Disease Word Learning subtest (CERAD-WL) immediate and delayed memory, the Animal fluency test (AFT), and the Digit Symbol Substitution Test (DSST). Test-specific and global cognition z-scores were calculated based on sample means and standard deviations. Multivariable linear regression models were applied to examine the association of quartiles of serum soluble alpha-klotho with test-specific and global cognition z-scores. Subgroup analysis was conducted by sex. The following covariates were included in the analysis- age, sex, race/ethnicity, education, depressive symptoms, smoking status, body mass index (BMI), physical activity, stroke, prevalent coronary heart disease, total cholesterol, and systolic blood pressure.

**Results:**

Serum soluble alpha-klotho level in the lowest quartile was associated with lower z-scores for DSST (beta [β] =-0.13, 95% confidence interval [CI]: -0.25, -0.01). For subgroup analysis, serum soluble alpha-klotho level in the lowest quartile was associated with lower z-scores for DSST (β=-0.16, 95% CI: -0.32, -0.003) and global cognition (β=-0.14, 95% CI: -0.28, -0.01) among female participants. No significant association was found among male participants.

**Conclusions:**

Lower serum soluble alpha-klotho level was associated with poorer cognitive functioning among older women.

